# Could age increase the strength of inverse association between ultraviolet B exposure and colorectal cancer?

**DOI:** 10.1186/s12889-021-11089-w

**Published:** 2021-07-05

**Authors:** Vidya Lakshmi Purushothaman, Raphael E. Cuomo, Cedric F. Garland, Timothy K. Mackey

**Affiliations:** 1 Department of Anesthesiology, University of California, San Diego, USA; 2 Global Health Policy Institute, San Diego, USA; 3 Division of Extended Studies, University of California, San Diego, USA; 4 Department of Family Medicine and Public Health, University of California, San Diego, USA

**Keywords:** Colorectal cancer, Ultraviolet B rays, UVB, Vitamin D, Aging, Crude incidence rates

## Abstract

**Background:**

Vitamin D has been identified as a potential protective factor in the development of colorectal cancer (CRC). We expect to see a stronger association of ultraviolet B (UVB) exposure and CRC crude rates with increasing age since chronic vitamin D deficiency leads to sustained molecular changes that increase cancer risk. The DINOMIT (disjunction, initiation, natural selection, overgrowth, metastasis, involution, and transition) model postulates various stages of cancer development due to vitamin D deficiency and the associated latency period. The purpose of this study is to examine this age-dependent inverse relationship globally.

**Methods:**

In this ecological study, a series of linear and polynomial regression tests were performed between country-specific UVB estimates adjusted for cloud cover and crude incidence rates of CRC for different age groups. Multiple linear regression was used to investigate the association between crude incidence rates of colorectal cancer and UVB estimate adjusting for urbanization, skin pigmentation, smoking, animal consumption, per capita GDP, and life expectancy. Statistical analysis was followed by geospatial visualization by producing choropleth maps.

**Results:**

The inverse relationship between UVB exposure and CRC crude rates was stronger in older age groups at the country level. Quadratic curve fitting was preferred, and these models were statistically significant for all age groups. The inverse association between crude incidence rates of CRC and UVB exposure was statistically significant for age groups above 45 years, after controlling for covariates.

**Conclusion:**

The age-dependent inverse association between UVB exposure and incidence of colorectal cancer exhibits a greater effect size among older age groups in global analyses. Studying the effect of chronic vitamin D deficiency on colorectal cancer etiology will help in understanding the necessity for population-wide screening programs for vitamin D deficiency, especially in regions with inadequate UVB exposure. Further studies are required to assess the need for adequate public health programs such as selective supplementation and food fortification.

**Supplementary Information:**

The online version contains supplementary material available at 10.1186/s12889-021-11089-w.

## Background

Colorectal cancer (CRC) is the third most common cancer globally with over 4 million prevalent cases. Nearly 2 million new cases of colorectal cancer were reported worldwide in 2018 [1]. It is the third most common cause of cancer in the United States with an estimated 460,714 cases in 2018 [1]. It is the second most common cause of death due to cancer worldwide and within the United States (880,000 deaths worldwide and fifty thousand deaths in the United States in 2018) [1]. The global burden of CRC is expected to increase by 60% to more than 2.2 million new cases and 1.1 million cancer deaths by 2030 [2]. The need for advanced prevention and treatment strategies has increased due to the need to reduce cancer morbidity and mortality for colorectal cancer.

Some of the risk factors linked to increased risk of developing CRC include obesity, sedentary lifestyle, consumption of high-fat, high-meat diets and calorie rich and fiber deficient food [3]. Apart from these known risk factors, inadequate vitamin D status as assessed by serum 25-hydroxyvitamin D (25(OH)D) concentration has also been identified as a potential risk factor in the pathogenesis of CRC. Vitamin D has been identified as a potential protective factor in the risk of developing CRC. Intake of 1000 IU/day of vitamin D is shown to be associated with 50% lower risk [4]. A meta-analysis using random effects model showed that the hazard ratio for mortality was lower with higher serum vitamin D status [5]. The results of this study suggested regular testing and restoration of vitamin D status to adequacy for lowering the mortality in colorectal cancer [5].

Vitamin D is a fat-soluble vitamin which has limited dietary sources and is predominantly obtained when exposed to ultraviolet B (UVB) radiation in sunlight [6]. Previtamin D_3_ is an intermediate product in the production of cholecalciferol. It is formed when UVB light of wavelengths between 280 and 315 nm from sunlight acts on 7-dehydrocholesterol present in the epidermal layers of the skin. It then converts by spontaneous isomerization into cholecalciferol which is converted into the active form of vitamin D through two-step hydroxylation [6]. Availability and exposure to UVB in sunlight is strongly correlated to the concentration of calcidiol and calcitriol levels in blood. UVB exposure and supplemental vitamin D both increase calcitriol in a dose-dependent fashion, [7] and increases in calcitriol have been shown to depend on baseline vitamin D status [8, 9]. In addition, a number of molecular factors may influence levels of serum 25(OH) D, including expression of the APOEε4 allele [10].

A recent study showed that most patients with a new diagnosis of CRC had deficient levels of serum 25(OH)D [11]. Better survival rates have been observed in patients with higher serum 25(OH) D concentrations compared to those with lower concentrations [12]. Reviews of ecological studies have shown evidence for the association between UVB–vitamin D–cancer to be convincing for several different types of cancer [13, 14]. However, not all studies have shown an increased cancer risk associated with inadequate circulating vitamin D levels. A Mendelian randomization study provided little evidence for the association of vitamin D and risk of several types of cancer [15].

The DINOMIT model (disjunction, initiation, natural selection, overgrowth, metastasis, involution, and transition) postulates that the anti-cancer effects of vitamin D can occur across these various phases of cancer etiology [16]. Vitamin D plays a protective role in all phases by protecting intercellular gap junctions through regulation of cadherins. Tight junctions prevent cells from separating and reduce the rate of cancer progression and metastasis. The DINOMIT model also postulates the involution of cancer through replenishment of vitamin D. Vitamin D deficiency’s effect on carcinogenesis is modeled as a function of time. With increasing age, the consequence of vitamin D deficiency accumulates longitudinally. Hence, the inverse epidemiological association between vitamin D status and incidence of colorectal cancer is expected to increase with age due to the chronicity of vitamin D deficiency. Since UVB exposure is strongly correlated to serum concentrations of 25(OH) D in the body, the strength of the inverse association between UVB estimate of a geographical area and the crude incidence rate of colorectal cancer can be studied to assess the longitudinal accumulation of carcinogenesis from vitamin D deficiency.

UVB exposure in a geographic area is affected by cloud cover, stratospheric ozone, altitude over sea level, skin pigmentation, number of hours spent indoors and type of clothing. Vitamin D production from UVB exposure may also be affected by air pollution and environmental chemicals [17]. Influential covariates that can affect CRC incidence include stratospheric ozone, diet, smoking prevalence, life expectancy, and wealth. The primary objective of this study to explore the effect of age on the inverse association between UVB exposure and CRC incidence, while adjusting for influential covariates. We hypothesize that the strength of the inverse association between UVB exposure and CRC incidence increases with age.

## Methods

### Study design

We conducted an ecological study assessing the age-dependent strength of inverse relationships between cloud cover-adjusted UVB exposure and incidence of CRC globally.

### Data sources

#### Primary outcome

The most recent age-stratified, country-specific crude rates of CRC worldwide were obtained from the Global Cancer (GLOBOCAN) database, using Cancer Today [1]. Cancer Today is a data visualization tool developed by the International Agency for Research on Cancer (IARC), a specialized cancer agency of World Health Organization (WHO). It provides estimates of the incidence, mortality, and prevalence of various cancers worldwide. Age-stratified crude incidence rates of CRC were available for 186 countries. Crude incidence rates were collected for the year 2018.

#### Primary predictor

Estimates for UVB (280–315 nm), adjusted for cloud cover and aerosols, were obtained from a visualization of April 2017 data from the National Aeronautics and Space Administration (NASA) EOS Aura spacecraft, available from a prior publication [18]. April was chosen as it was the closest month visualized to the spring equinox in 2017 (which occurred on March 20th). This image was processed using ArcGIS geospatial software to provide a mean UVB estimate for each country. Specifically, efforts were made to remove country borders, after which geospatial processing algorithms leveraged raster transformation and zonal statistics functions. The output of geoprocessing was a unitless measure of UVB between the lowest and highest possible values of 0 and 255, respectively.

#### Covariates

The objective of this study is to use UVB estimates to better understand whether low levels of vitamin D may be among the risk factors for development of CRC. As a number of factors influence the development of CRC, a wide variety of covariates were included in multiple regression analyses. Stratospheric ozone data was obtained from the NASA satellite instrument packages [19]. Data on life expectancy and GDP per capita (at purchasing power parity [PPP]) by country for those born in 2010 were provided by the World Bank [20]. GDP at PPP is nominal gross domestic product converted to international dollars using purchasing power parity rates [20]. Data on pigmentation by country was available from published literature [21]. Data on urbanization percent (urban population fraction) by country were available from a previous publication [22]. Data on smoking prevalence was collected from Global Health Data Exchange (GHDx) from the Institute for Health Metrics and Evaluation (IHME) [23]. Daily smoking prevalence was the percentage of the national population that smokes daily. Data on animal meat consumption (kilogram/capita/year) were available from the Food and Agricultural Organization of the United Nations (FAO) [24]. Data for all covariates were collected for the year 2010 (8 years prior to the incidence data).

Data for country-specific modeled serum 25(OH) D were available from a previous publication [25]. Modeling of the estimated serum 25(OH) D included measured levels of serum 25(OH) D during winter as a dependent variable, UVB irradiance was included as an independent variable and skin pigmentation as a covariate from 28 publications [25]. For countries where actual measured levels of serum 25(OH) D were not available, a prediction equation was obtained using the regression coefficients of the models for the countries with measured 25(OH) D levels [25].

### Statistical analysis

Age-stratified crude incidence rates of CRC were available for 185 countries. However, data for adjusted UVB estimates were available for only 166 countries (a list of excluded countries is provided in Appendix A). Data for all covariates were available for 148 countries. Primary statistical analyses were conducted for 166 countries and multiple linear regression was employed for 148 countries (a list of countries excluded from the multiple linear regression model is provided in Appendix B). Spearman’s correlation test was used to analyze the association between adjusted UVB estimates and country-specific crude incidence rates of CRC for every age group (0–14, 15–29, 30–44, 45–59, 60–74, >/= 75 years of age). A series of simple linear regression tests were performed followed by polynomial regressions between country-specific UVB estimates adjusted for cloud cover and crude incidence rates of CRC for different age groups. A better curve fit was obtained using a quadratic term. Labelled scatter plots with polynomial trend lines were plotted for each quadratic model (a list of the label codes for the names of countries (ISO 3166 standard) is provided in Appendix C). Unadjusted quadratic models for southern hemisphere countries and northern hemisphere countries were analyzed for over 75 years of age. In addition, a subset of countries had estimates available from high-quality registry data in the IARC’s CI5*plus* database, so a separate linear model was computed only using estimates from these countries. As sample size was relatively limited for this sub-analysis, a univariate model of the relationship between UVB and crude CRC rates above age 75 was assembled.

Multiple linear regression was used to investigate the relationship between crude incidence rates of colorectal cancer and cloud cover-adjusted UVB controlling for stratospheric ozone, urbanization, skin pigmentation, smoking, animal consumption, per capita GDP, and life expectancy. A smoking covariate was also included in the age group of 0–14 years to capture the impact of second-hand smoke on adolescents. Adjusted models for southern hemisphere countries and northern hemisphere countries were analyzed for over 75 years of age. A series of multiple linear regression models were employed to study the association between crude incidence rates of colorectal cancer and modeled 25(OH) D, while controlling for urbanization, smoking, animal consumption, per capita GDP and life expectancy. UVB irradiance and skin pigmentation were taken into account in the modeled 25(OH) D values. A *p*-value of < 0.05 was considered statistically significant for all analyses. Statistical analyses were performed using R version 3.6.0. Choropleth maps of country-specific CRC incidence for every age group were produced in which countries were color coded according to incidence rates. All choropleth maps were produced using QGIS software.

## Results

### Main results

**Fig. 1:**
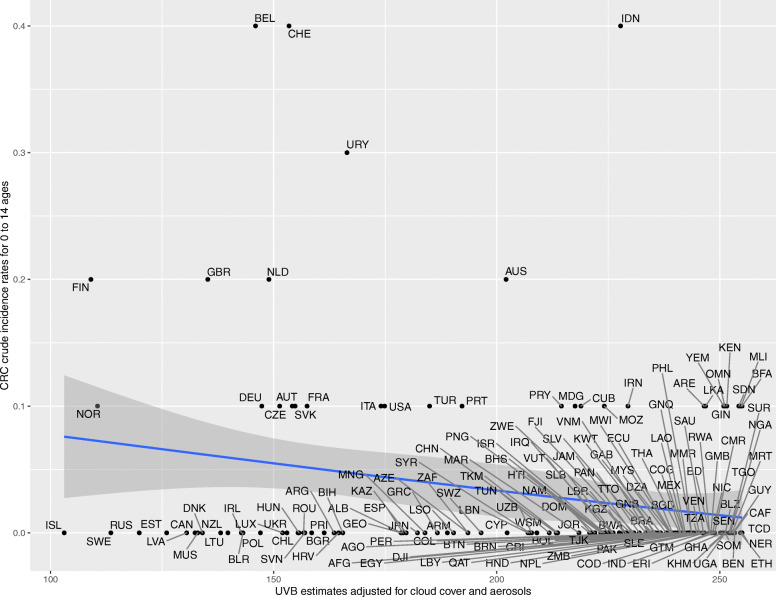
Estimated crude incidence rates of CRC in 0–14 years of age by UVB estimates, 2018

**Fig. 2:**
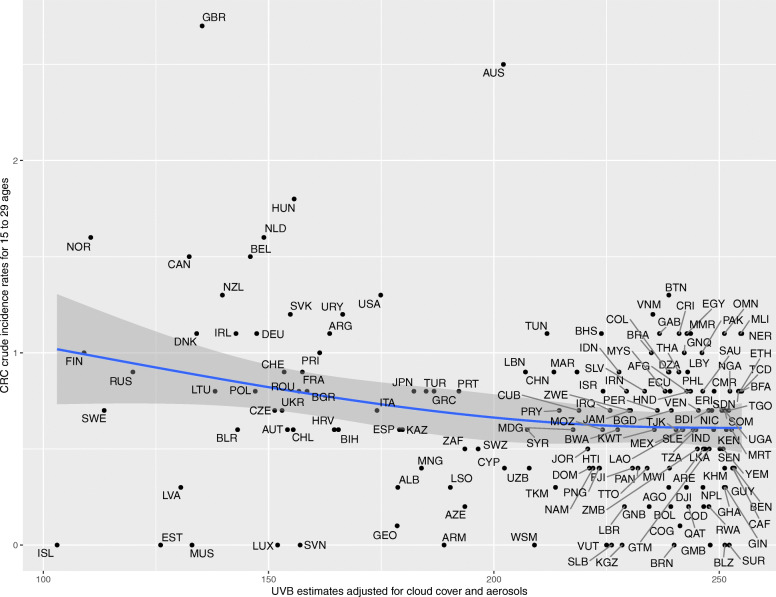
Estimated crude incidence rates of CRC in 15–29 years of age by UVB estimates, 2018

**Fig. 3:**
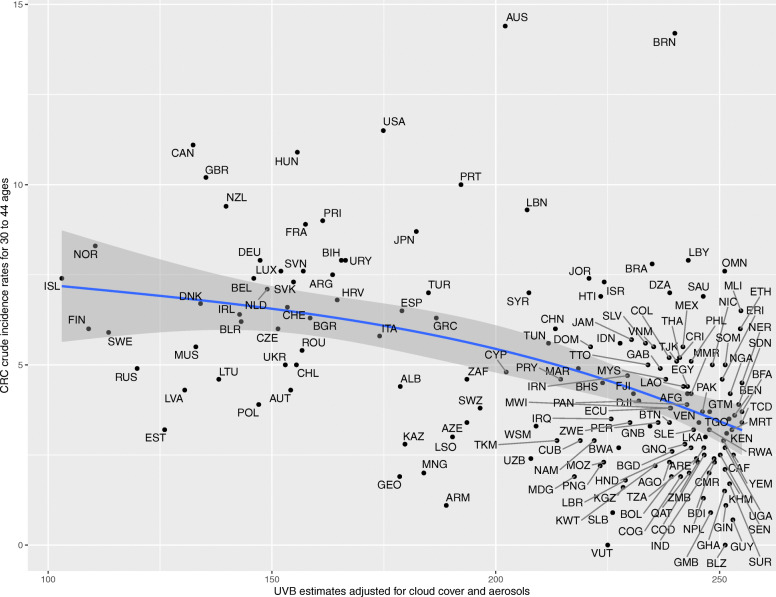
Estimated crude incidence rates of CRC in 30–44 years of age by UVB estimates, 2018

**Fig. 4:**
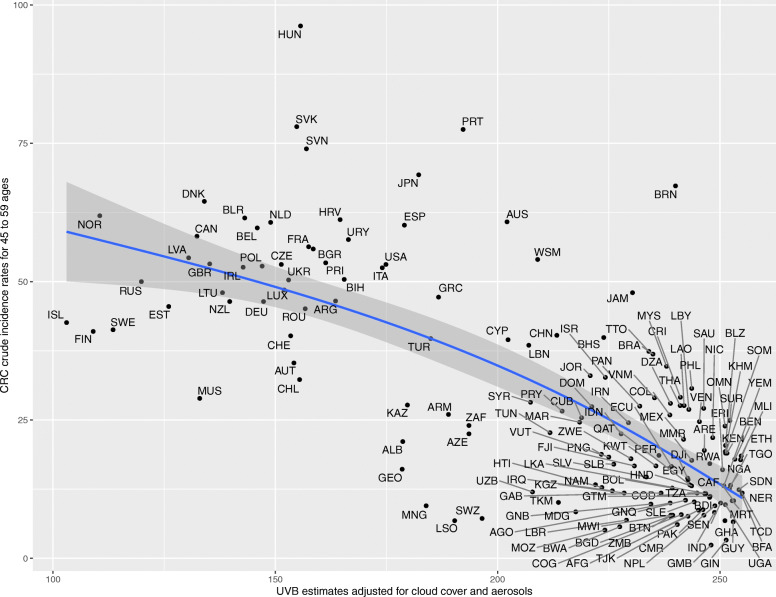
Estimated crude incidence rates of CRC in 45–59 years of age by UVB estimates, 2018

**Fig. 5:**
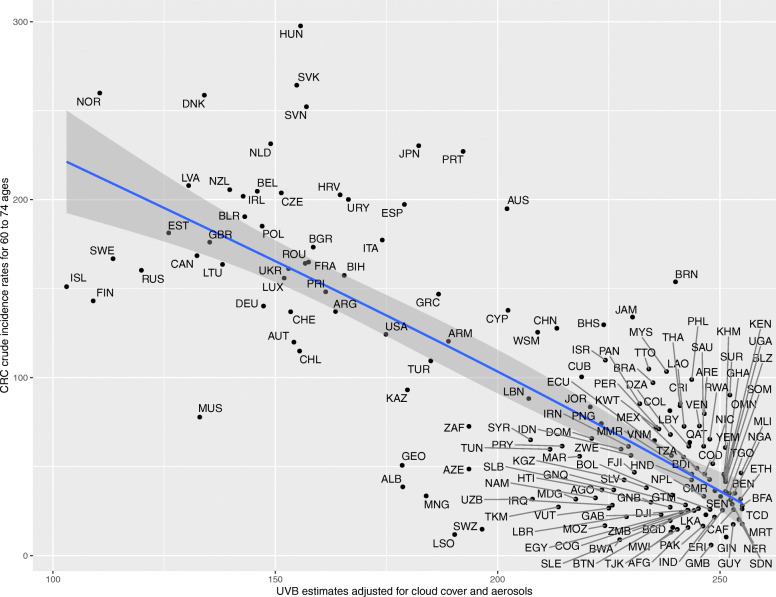
Estimated crude incidence rates of CRC in 60–74 years of age by UVB estimates, 2018

**Fig. 6:**
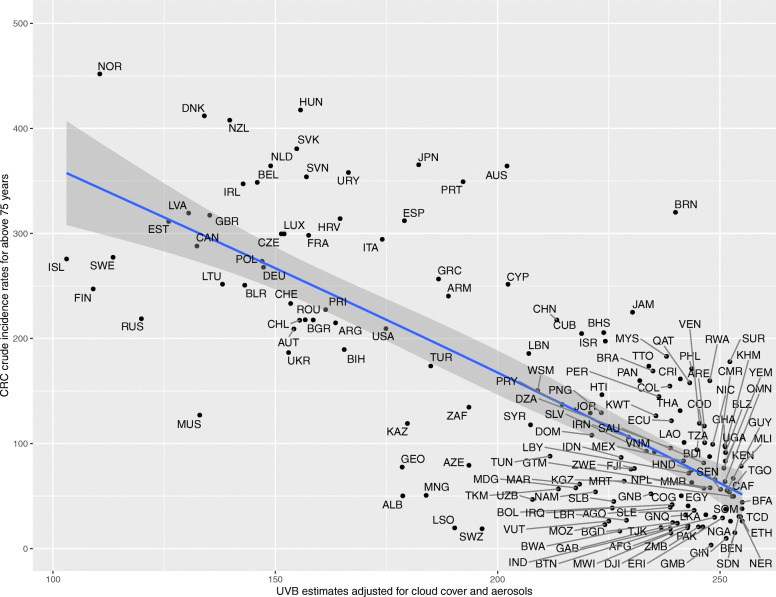
Estimated crude incidence rates of CRC in >/=75 years of age by UVB estimates, 2018

Upon generating scatter plots for polynomial regression of adjusted UVB with crude incidence of CRC for each age group, an increasing trend was seen in the strength of the inverse relationship between incidence of CRC and adjusted UVB with increasing age (Figs. 1, 2, 3, 4, 5 and 6). Polynomial trend lines provided a better fit compared to linear fit trend lines. Spearman’s rank correlation tests between incidence of CRC and UVB for each age group showed negative correlations of increasing strength with increasing age (Table 1).

**Table 1:** Correlation between colorectal cancer crude incidence rate and ultraviolet B estimate for every age group

Age groups	Spearman’s rank correlation rho	*p*-value
0–14 years	− 0.16	0.05
15–29 years	− 0.16	0.049
30–44 years	− 0.50	< 0.001
45–59 years	− 0.68	< 0.001
60–74 years	−0.70	< 0.001
>/= 75 years	−0.69	< 0.001

Table 2 illustrates the results of linear regression tests between cloud cover adjusted UVB and CRC crude incidence rate for every age group.

**Table 2:** UVB estimate in association with crude incidence rate of CRC using linear regression

Age groups	Regression Coefficient	*p*-value	R^2^
0–14 years	< 0.001	0.002	0.06
15–29 years	− 0.002	0.003	0.05
30–44 years	− 0.03	< 0.001	0.23
45–59 years	− 0.34	< 0.001	0.54
60–74 years	− 1.28	< 0.001	0.62
>/= 75 years	−2.03	< 0.001	0.59

Polynomial regression models for each age group between adjusted UVB estimates and CRC crude incidence rate showed a stronger inverse association for older age groups compared to younger age groups (Table 3). The overall *p*-value of the polynomial model was statistically significant for every age group. Also, the overall R^2^ of the polynomial model increased with age and the highest R^2^ (0.62) was obtained for 64–75 years of age. The overall R^2^ value of the polynomial model for countries in northern hemisphere for over 75 years of age was higher than that for countries in southern hemisphere before adjusting for covariates (Appendix Tables 5 and 6).

**Table 3:** UVB estimate in association with crude incidence rate of CRC using polynomial regression

Age groups	Regression Coefficient		P-value (overall)	R^2^ (overall)
	UVB	UVB^2		
0–14 years	< 0.001	< 0.001	0.01	0.045
15–29 years	− 0.007	< 0.001	0.01	0.04
30–44 years	0.04	< 0.001	< 0.001	0.24
45–59 years	0.27	−0.002	< 0.001	0.55
60–74 years	−0.51	− 0.002	< 0.001	0.62
>/= 75 years	− 1.78	−0.002	< 0.001	0.58

### Other analyses

In the multiple linear regression model, UVB was inversely associated with crude incidence rates of colorectal cancer for all age groups above 45 years, after controlling for covariates (*p*-value < 0.05) (Table 4*;* Appendix Tables 7, 8, 9, 10 and 11). The highest adjusted model R^2^ (0.71) was obtained for 64–75 years of age (Appendix Table 11) and over 75 years of age (Table 4). The tables for the other age groups are provided in the appendix (Appendix Tables 7, 8, 9, 10 and 11). After adjusting for covariates, the model R^2^ value of the polynomial model for countries in southern hemisphere for over 75 years of age was higher than that for countries in northern hemisphere (Appendix Tables 12 and 13). Also, univariate regression analysis for CRC rates above age 75, only among the 29 countries with high-quality cancer registries available from the CI5*plus* database, exhibited a statistically significant association with UVB (*p* <  0.001; Appendix Table 14). The overall models were statistically significant with a *p*-value < 0.001 for age groups above 45 years. The association between UVB estimates and crude incidence rates of CRC was not statistically significant in age groups below 45 years, after controlling for covariates.

**Table 4:** UVB in association with crude incidence of CRC: over 75 years of age, controlling for covariates

Covariate	Regression Coefficient	Standard Error	*t*	*p*-value
UV B estimate ^a^	− 1.02	0.21	− 4.87	< 0.001
Stratospheric Ozone^b^	−0.96	0.21	−0.68	0.49
Urbanization^c^	0.28	0.35	0.83	0.41
Pigmentation^d^	−15.11	9.83	−1.54	0.13
GDP^e^	0.001	< 0.001	2.82	0.006
Life Expectancy^f^	0.005	0.006	0.81	0.42
Smoking^g^	0.49	0.92	0.54	0.59
Animal Consumption^h^	0.76	0.31	2.47	0.02
Intercept	329.3	67.29	4.89	< 0.001

(*Model R*^*2*^ *= 0.71; p <  0.001);* ^a^NASA TOMS Satellite package. ^b^Beckman et al. ^c^Christenson E et al. [22]. ^d^Jablonski et al. [21]. ^e,f^The World Bank. ^g^Institute for Health Metrics and Evaluation (IHME). ^h^The Food and Agricultural Organization of the United Nations (FAO)

According to the multiple linear regression models, the inverse association between modeled 25(OH) D and crude incidence rates of CRC was statistically significant in age groups 60–74 years, 75 years and above (Appendix Tables 15, 16, 17, 18, 19 and 20). The association was marginally significant in the age groups of 45–59 years. In age groups below 45 years, the association was not statistically significant.

Choropleth maps produced using QGIS visualized the distribution of colorectal cancer worldwide. Supplementary file Figures S1, S2, S3, S4 and S5 illustrate the distribution of CRC in different countries for every age group.

## Discussion

### Key results

This study aims at assessing the strength of the inverse relationship between UVB exposure and CRC incidence with increasing age. The DINOMIT model [16] proposes an explanation of how vitamin D deficiency increases the risk of developing colorectal cancer. However, it is expected to take years for these phases to occur, and hence we expect increasing age to have a major role in explaining the inverse relationship between UVB estimates and incidence of colorectal cancer. Thus, older age groups can be expected to have a stronger inverse association between vitamin D status and crude incidence rate of colorectal cancer. Though there is mixed evidence for this inverse association, our study aims at taking into consideration the effect of age on this association. In this country-specific analysis, we have shown an increasing trend in the strength of the inverse association between adjusted UVB estimates and crude incidence rate of colorectal cancer as age increases. The proportion of variability in the outcome (crude incidence rate of CRC) explained by the adjusted UVB estimate also increased with age. This study assesses the age-dependent inverse association between vitamin D status and incidence of colorectal cancer globally. This is the first study to the authors’ knowledge to have explored the age-related effect in this inverse association. UVB estimates decrease with increasing latitude, and higher incidence of colorectal cancer has been reported at higher latitudes [26]. Another recent study mentions low vitamin D status as a possible explanation to higher incidence rates of colon cancer in cold countries (higher latitudes) [27]. This study demonstrates a significant inverse association between UVB exposure and CRC incidence in all age groups. Age-related differences in vitamin D status have been observed in the regions of Asia/Pacific and Middle East/Africa [28] and reduced vitamin D status with increasing age has been reported in previous studies [29]. Also, vitamin D deficiency has been observed across all age groups globally including countries with low latitude [30]. UVB exposure is strongly correlated with serum 25(OH) D levels and previous studies have shown significant associations between 25(OH) D levels and overall CRC incidence [31]. Photosynthesized vitamin D released from erythemal solar radiation to the skin has been found to have a greater effect on serum 25(OH) D levels than dietary vitamin D ingestion [32]. The tissue stores of cholecalciferol which are obtained through exposure to UVB radiation help in sustaining serum 25(OH) D levels [32].

### Interpretation

Previous ecological studies have reported an inverse association between UVB exposure and incidence as well as mortality of various cancers, including colon cancer [33, 34]. In one of the prior ecological studies, a significant inverse association was observed for colon cancer (among ten other cancers), and the relative risk of colon cancer incidence related to solar UVB exposure was found to be 1.11 in males and 1.14 in females [33]. In another ecological study, inverse associations with UVB were found for 15 different cancers, including colon cancer [34]. The standardized regression coefficient for age-adjusted mortality rates of colon cancer versus UVB irradiance was found to be − 0.71 (*p* <  0.001) for males and − 0.76 (p <  0.001) for females. However, not all ecological studies have been able to demonstrate a significant inverse association between UV exposure and colon cancer [35]. The association between UVB exposure and global incidence of colorectal cancer was first analyzed in an ecological study [26] where simple linear regression and multiple linear regression methods were used to study the inverse association between UVB exposure and incidence of CRC. In this study, the age-adjusted crude incidence rates of colorectal cancer were higher at latitudes distant from the equator (R^2^ = 0.50, *p* <  0.001) [26]. In the adjusted model of that study, UVB exposure (adjusted for cloudiness) was inversely associated with age adjusted CRC crude incidence rates (*p* = 0.01), after controlling for covariates [26]. However, age-dependent strength of the inverse association between UVB exposure and colorectal cancer was not explored in that study [26].

Various studies have demonstrated the effect of diet on risk of colorectal cancer. Increased consumption of red meat and total meat were associated with higher risk of developing colorectal cancer in a study which analyzed data from a Japanese cohort [36]. Also, intake of fruits and vegetables have shown to have a protective effect against cancer [37]. Results from other studies suggest that changes in dietary pattern, specifically with increased meat consumption, can increase the risk of developing CRC [38]. A recent study observed highest number and proportion of diet-related cases for colorectal cancer [39]. Smoking is known to increase the risk of both colon and rectal cancer, with a stronger association for rectal cancer [40, 41]. The duration of smoking had a significant association with the risk of colorectal cancer [42].

A nested case-control study from the Women’s Health Study (WHS) found a significant inverse association between pre-diagnostic 25(OH) D levels and risk of CRC [43]. This case-control study with 274 controls and 274 colorectal cases observed a significant inverse association between plasma vitamin D and odds of colorectal cancer in multivariable adjusted logistic regression models [43]. MEG3 (non-coding RNA maternally expressed gene) functions as a tumor suppressor in CRC by regulating the activity of clusterin, which is stimulated by the binding of vitamin D receptor to its promoter [44]. Meta-analysis of the relationship between serum 25(OH) D and mortality of patients with colorectal cancer has shown that higher serum 25(OH) D was associated with lower mortality of patients with colorectal cancer [5]. Mortality rates were also decreased in summertime, where UVB wavelengths of solar radiation are more available [45].

The main strength of this study is the novelty of assessing the age-dependent inverse relationship between UVB exposure and CRC incidence. The unadjusted analysis included 166 countries in comparison to 139 countries in a previous study [26]. The results of this analysis are in line with the previous study [26] in having obtained a significant inverse association between UVB exposure and incidence of colorectal cancer. As with prior analyses, this analysis employed multiple linear regression to account for other risk-modifying factors. UVB estimates were significantly associated with the risk of colorectal cancer in age groups over 45 years after adjusting for covariates. These findings are consistent with other studies which have found significantly different risk factors for individuals receiving a diagnosis of CRC prior to age 50, compared to those receiving a diagnosis after age 50 [46]. We suggest that several risk factors for later-age development of CRC may derive from chronic exposures, and we suggest that vitamin D deficiency is among these. The significant increase in the strength of this inverse association with age was observed in the analysis, as hypothesized. Also, the proportion of variation in the age-specific crude incidence rates due to UVB exposure (R^2^) increased consistently with age.

### Limitations

Data for all variables that were included in the multiple linear regression were available for only 148 countries out of the 185 countries for which CRC crude incidence rate data were available. However, the excluded countries account for approximately 3% of global population. Also, the inherent limitations of the data used in this study include use of neighboring country’s CRC estimate in some cases of unavailability, as well as some uses of hospital-based data instead of population/registry-based data. Furthermore, countries which lacked data for UVB estimates were mostly countries with lower per capita income and limited access to healthcare, which were also closer to equator with high UVB exposure. Exclusion of these countries from the study could have reduced the strength of associations. Though laboratory research and studies on individuals have produced evidence validating the influence of UVB on serum 25(OH) D, we note that UVB is an imperfect proxy measure of 25(OH) D status. National mean 25(OH) D concentration depends on a large number of factors. These include UVB irradiation, cloud cover, skin pigmentation, and urbanization, which are factors which were included in this study’s multivariable modeling. However, additional factors, including vitamin D supplementation, clothing cover area, altitude over sea level, air pollution and environmental chemicals are also likely to be relevant, but were not included in this study due to limited availability of data or to preserve model parsimony.

### Generalizability

The results of the study cannot be applied directly at the level of individuals due to ecological fallacy. However, the study findings do reveal a significant effect of age on the inverse association between UVB exposure and colorectal cancer incidence rates. The multivariate models with modeled 25(OH) D had lower R^2^ values compared to those with the UVB estimates adjusted for cloud cover, reducing the proportion of variability in the crude incidence of CRC explained by vitamin D levels. Also, adjusted UVB estimate was statistically significant in the age group of 45–60 years, whereas modeled 25(OH) D was only marginally significant although both the covariates retained statistical significance in age groups above 60 years. Though our study used the modeled 25(OH) D data calculated from 28 publications, there was a mean difference of 5.26 nmol/L between the values used in the study and the published annual values of 25(OH)D [28, 29].

## Conclusion

Ecological studies help in generating novel, relevant hypothesis that may help in identifying causal relationships that can be further explored through studies on individuals. This study supports the need for adequate public health programs to avoid vitamin D inadequacy at national and global levels, whether through screening those at risk, through selective supplementation, or through population-based measures such as food fortification. Future studies can aim at identifying the cancer types which show significant improvement with vitamin D supplementation. Studying the association between chronic vitamin D deficiency and CRC incidence will help in understanding the necessity for population-wide screening programs for vitamin D deficiency, especially in regions with inadequate UVB exposure. These programs may help decrease risk of CRC, as well as other cancers whose risk is associated with vitamin D deficiency, for high-risk populations whose vitamin D deficiency has been especially chronic.

### Supplementary Information

**Additional file 1: Figure S1.** Colorectal cancer crude incidence rates, 15–29 years of age, all races, both sexes, 2018.

**Additional file 2: Figure S2.** Colorectal cancer crude incidence rates, 30–44 years of age, all races, both sexes, 2018.

**Additional file 3: Figure S3.** Colorectal cancer crude incidence rates, 45–59 years of age, all races, both sexes, 2018.

**Additional file 4: Figure S4.** Colorectal cancer crude incidence rates, 60–74 years of age, all races, both sexes, 2018.

**Additional file 5: Figure S5.** Colorectal cancer crude incidence rates, >/=75 years of age, all races, both sexes, 2018.

## Data Availability

The following data sources (open to public access) were used to collect data for this study: 1. Colorectal cancer incidence rates: Cancer Today, Ferlay J, Ervik M, Lam F, Colombet M, Mery L, Piñeros M, Znaor A, Soerjomataram I, Bray F (2018). Global Cancer Observatory: Cancer Today. Lyon, France: International Agency for Research on Cancer. Available from: https://gco.iarc.fr/today 2. Estimates for UVB (280–315 nm), adjusted for cloud cover and aerosols: Beckmann M, Václavík T, Manceur AM, Šprtová L, von Wehrden H, Welk E, et al. glUV: a global UV-B radiation data set for macroecological studies. Tatem A, editor. Methods Ecol Evol [Internet]. 2014 Apr 1 [cited 2020 Apr 1];5 (4):372–83. Available from: http://doi.wiley.com/10.1111/2041-210X.12168 3. Stratospheric ozone data: Earthdata [Internet]. Available from: https://earthdata.nasa.gov/ 4. Data on life expectancy and GDP per capita (at purchasing power parity [PPP]): World Bank Open Data; https://data.worldbank.org/ 5. Pigmentation data: Jablonski NG, Chaplin G. The evolution of human skin coloration. J Hum Evol 2000; 39:57–106; PMID:10896812; 10.1006/jhev.2000.0403. 6. Urbanization data: Christenson E, Elliott M, Banerjee O, Hamrick L, Bartram J. Climate-related hazards: a method for global assessment of urban and rural population exposure to cyclones, droughts, and floods. Int J Environ Res Public Health. 2014;11 (2):2169–2192. 7. Smoking prevalence: Institute for Health Metrics and Evaluation (IHME). Global Smoking Prevalence and Cigarette Consumption 1980–2012. Seattle, United States: Institute for Health Metrics and Evaluation (IHME), 2014. 8. Data on animal meat consumption: The Food and Agricultural Organization of the United Nations. http://www.fao.org/faostat/en/#data/FBS. The datasets used and/or analyzed during the current study can be accessed on GitHub repository https://github.com/ghpi2021/vitD_age

## Appendix

**Table 1:** UVB estimate in association with crude incidence rate of CRC using polynomial regression for countries in northern hemisphere

Age groups	Regression Coefficient		P-value (overall)	R^2^ (overall)
	UVB	UVB^2		
>/= 75 years	− 0.49	− 0.004	< 0.001	0.62

**Table 2:** UVB estimate in association with crude incidence rate of CRC using polynomial regression for countries in southern hemisphere

Age groups	Regression Coefficient		P-value (overall)	R^2^ (overall)
	UVB	UVB^2		
>/= 75 years	− 2.26	< 0.001	0.002	0.36

**Table 3:** UVB in association with crude incidence of CRC: 0–14 years of age, controlling for covariates

Covariate	Regression Coefficient	Standard Error	*t*	*p*-value
UV B estimate^a^	< 0.001	< 0.001	−0.74	0.46
Stratospheric Ozone^b^	< 0.001	< 0.001	3.56	< 0.001
Urbanization^c^	< 0.001	< 0.001	0.30	0.77
Pigmentation^d^	−0.006	0.01	− 0.51	0.61
GDP^e^	< 0.001	< 0.001	2.19	0.03
Life Expectancy^f^	< 0.001	< 0.001	0.21	0.83
Smoking^g^	< 0.001	0.001	−0.78	0.43
Animal Consumption^h^	< 0.001	< 0.001	− 1.48	0.14
Intercept	0.07	0.08	−0.87	0.39

*Model R*^*2*^ *= 0.15; p <  0.001;* ^a^NASA TOMS Satellite package. ^b^Beckman et al. ^c^Christenson E et al. [22]. ^d^Jablonski et al. [21]. ^e,f^The World Bank. ^g^Institute for Health Metrics and Evaluation (IHME). ^h^The Food and Agricultural Organization of the United Nations (FAO)

**Table 4:** UVB in association with crude incidence of CRC: 15–29 years of age, controlling for covariates

Covariate	Regression Coefficient	Standard Error	*t*	*p*-value
UV B estimate^a^	0.002	0.001	1.21	0.23
Stratospheric Ozone^b^	0.001	< 0.001	1.50	0.14
Urbanization^c^	0.003	0.002	1.41	0.16
Pigmentation^d^	−0.23	0.06	−3.46	< 0.001
GDP^e^	< 0.001	< 0.001	−0.60	0.55
Life Expectancy^f^	< 0.001	< 0.001	−0.16	0.87
Smoking^g^	−0.001	0.006	−1.91	0.10
Animal Consumption^h^	< 0.001	0.002	0.41	0.06
Intercept	0.35	0.44	0.80	0.68

*Model R*^*2*^ *= 0.17; p <  0.001;* ^a^NASA TOMS Satellite package. ^b^Beckman et al. ^c^Christenson E et al. [22]. ^d^Jablonski et al. [21]. ^e,f^The World Bank. ^g^Institute for Health Metrics and Evaluation (IHME). ^h^The Food and Agricultural Organization of the United Nations (FAO)

**Table 5:** UVB in association with crude incidence of CRC: 30–44 years of age, controlling for covariates

Covariate	Regression Coefficient	Standard Error	*t*	*p*-value
UV B estimate^a^	< 0.001	0.007	−0.13	0.90
Stratospheric Ozone^b^	0.007	0.005	1.52	0.13
Urbanization^c^	0.02	0.01	1.37	0.18
Pigmentation^d^	−0.70	0.32	−2.15	0.03
GDP^e^	< 0.001	< 0.001	2.56	0.01
Life Expectancy^f^	< 0.001	< 0.001	0.53	0.60
Smoking^g^	−0.03	0.03	−1.27	0.21
Animal Consumption^h^	0.01	0.01	1.13	0.26
Intercept	2.85	2.17	1.31	0.19

*Model R*^*2*^ *= 0.42; p <  0.001;* ^a^NASA TOMS Satellite package. ^b^Beckman et al. ^c^Christenson E et al. [22]. ^d^Jablonski et al. [21]. ^e,f^The World Bank. ^g^Institute for Health Metrics and Evaluation (IHME). ^h^The Food and Agricultural Organization of the United Nations (FAO)

**Table 6:** UVB in association with crude incidence of CRC: 45–59 years of age, controlling for covariates

Covariate	Regression Coefficient	Standard Error	*t*	*p*-value
UV B estimate^a^	−0.14	0.04	−3.52	< 0.001
Stratospheric Ozone^b^	0.01	0.03	0.39	0.70
Urbanization^c^	−0.01	0.07	−0.17	0.86
Pigmentation^d^	−2.14	1.83	−1.17	0.24
GDP^e^	< 0.001	< 0.001	1.88	0.06
Life Expectancy^f^	0.002	0.001	1.52	0.13
Smoking^g^	0.37	0.17	2.16	0.03
Animal Consumption^h^	0.20	0.06	3.42	< 0.001
Intercept	40.76	12.55	3.25	0.002

*Model R*^*2*^ *= 0.67; p <  0.001;* ^a^NASA TOMS Satellite package. ^b^Beckman et al. ^c^Christenson E et al. [22]. ^d^Jablonski et al. [21]. ^e,f^The World Bank. ^g^Institute for Health Metrics and Evaluation (IHME). ^h^The Food and Agricultural Organization of the United Nations (FAO)

**Table 7:** UVB in association with crude incidence of CRC: 60–74 years of age, controlling for covariates

Covariate	Regression Coefficient	Standard Error	*t*	*p*-value
UV B estimate ^a^	−0.69	0.13	−5.35	< 0.001
Stratospheric Ozone^b^	−0.02	0.09	−0.19	0.85
Urbanization^c^	0.05	0.22	0.23	0.82
Pigmentation^d^	−7.84	6.05	−1.29	0.20
GDP^e^	< 0.001	< 0.001	1.97	0.051
Life Expectancy^f^	0.004	0.004	0.10	0.33
Smoking^g^	0.95	0.57	1.67	0.10
Animal Consumption^h^	0.48	0.19	2.52	0.01
Intercept	200.9	41.45	4.85	< 0.001

*Model R*^*2*^ *= 0.71; p <  0.001*; ^a^NASA TOMS Satellite package. ^b^Beckman et al. ^c^Christenson E et al. [22]. ^d^Jablonski et al. [21]. ^e,f^The World Bank. ^g^Institute for Health Metrics and Evaluation (IHME). ^h^The Food and Agricultural Organization of the United Nations (FAO)

**Table 8:** UVB in association with crude incidence of CRC: over 75 years of age for countries in northern hemisphere after controlling for covariates

Covariate	Regression Coefficient	Standard Error	*t*	*p*-value
UV B estimate^a^	−1.43	0.25	−5.62	< 0.001
Stratospheric Ozone^b^	−0.10	0.15	−0.67	0.51
Urbanization^c^	0.10	0.38	0.26	0.80
Pigmentation^d^	12.94	12.53	1.03	0.30
GDP^e^	0.002	< 0.001	3.10	0.003
Life Expectancy^f^	0.009	0.008	1.11	0.27
Smoking^g^	1.41	0.95	1.50	0.14
Animal Consumption^h^	0.83	0.32	2.57	0.01
Intercept	359.70	71.06	5.06	< 0.001

*Model R*^*2*^ *= 0.72; p <  0.001;* ^a^NASA TOMS Satellite package. ^b^Beckman et al. ^c^Christenson E et al. [22]. ^d^Jablonski et al. [21]. ^e,f^The World Bank. ^g^Institute for Health Metrics and Evaluation (IHME). ^h^The Food and Agricultural Organization of the United Nations (FAO)

**Table 9:** UVB in association with crude incidence of CRC: over 75 years of age for countries in southern hemisphere after controlling for covariates

Covariate	Regression Coefficient	Standard Error	*t*	*p*-value
UV B estimate^a^	−1.18	0.47	−2.52	0.02
Stratospheric Ozone^b^	0.57	0.36	1.59	0.13
Urbanization^c^	0.53	0.78	0.67	0.51
Pigmentation^d^	−89.49	24.56	−3.64	0.002
GDP^e^	0.002	0.003	0.82	0.43
Life Expectancy^f^	0.02	0.01	1.73	0.10
Smoking^g^	−3.53	2.64	−1.34	0.20
Animal Consumption^h^	−0.82	1.02	−0.81	0.43
Intercept	475.41	179.95	2.54	0.02

*Model R*^*2*^ *= 0.81; p <  0.001;* ^a^NASA TOMS Satellite package. ^b^Beckman et al. ^c^Christenson E et al. [22]. ^d^Jablonski et al. [21]. ^e,f^The World Bank. ^g^Institute for Health Metrics and Evaluation (IHME). ^h^The Food and Agricultural Organization of the United Nations (FAO)

**Table 10:** UVB estimate in association with crude incidence rate of CRC using linear regression among countries with high-quality registry data in the CI5 *plus* database

Age groups	Regression Coefficient	*p*-value	R^2^
>/= 75 years	− 1.36	< 0.001	0.35

**Table 11:** Modeled 25(OH) D in association with incidence of CRC in 0–14 years of age, controlling for covariates

Covariate	Regression Coefficient	Standard Error	*t*	*p*-value
Modeled 25(OH) D, ng/ml^a^	< 0.001	< 0.001	−0.49	0.63
Urbanization^b^	< 0.001	< 0.001	0.53	0.60
GDP^c^	< 0.001	< 0.001	2.84	0.005
Life Expectancy^d^	< 0.001	< 0.001	−0.07	0.95
Smoking^e^	< 0.001	0.001	0.51	0.61
Animal Consumption^f^	< 0.001	< 0.001	−1.54	0.13
Intercept	0.02	0.04	0.50	0.62

*Model R*^*2*^ *= 0.06; p = 0.02;* ^a^Mohr S, B, Gorham E, D, Garland C, F, Grant W, B, Garland F, C: Low Ultraviolet B and Increased Risk of Brain Cancer: An Ecological Study of 175 Countries. Neuroepidemiology 2010; 35:281–290. doi: 10.1159/000314350. ^b^Christenson E et al. [22]. ^c,d^ The World Bank. ^e^Institute for Health Metrics and Evaluation (IHME). ^f^ The Food and Agricultural Organization of the United Nations (FAO)

**Table 12:** Modeled 25(OH) D in association with incidence of CRC in 15–29 years of age, controlling for covariates

Covariate	Regression Coefficient	Standard Error	*t*	*p*-value
Modeled 25(OH) D, ng/ml^a^	−0.004	0.004	−1.16	0.25
Urbanization^b^	0.005	0.002	2.01	0.046
GDP^c^	< 0.001	< 0.001	0.26	0.79
Life Expectancy^d^	< 0.001	< 0.001	−0.39	0.69
Smoking^e^	−0.003	0.006	−0.58	0.56
Animal Consumption^f^	< 0.001	0.002	0.37	0.71
Intercept	0.58	0.25	2.29	0.02

*Model R*^*2*^ *= 0.10; p = 0.002;* ^a^Mohr S, B, Gorham E, D, Garland C, F, Grant W, B, Garland F, C: Low Ultraviolet B and Increased Risk of Brain Cancer: An Ecological Study of 175 Countries. Neuroepidemiology 2010; 35:281–290. doi: 10.1159/000314350. ^b^Christenson E et al. [22]. ^c,d^ The World Bank. ^e^Institute for Health Metrics and Evaluation (IHME). ^f^ The Food and Agricultural Organization of the United Nations (FAO)

**Table 13:** Modeled 25(OH) D in association with incidence of CRC in 30–44 years of age, controlling for covariates

Covariate	Regression Coefficient	Standard Error	*t*	*p*-value
Modeled 25(OH) D, ng/ml^a^	−0.02	0.02	−1.23	0.22
Urbanization^b^	0.02	0.01	1.63	0.11
GDP^c^	< 0.001	< 0.001	3.20	0.002
Life Expectancy^d^	< 0.001	< 0.001	0.25	0.80
Smoking^e^	< 0.001	0.03	0.03	0.98
Animal Consumption^f^	0.01	0.01	1.15	0.25
Intercept	3.14	1.25	2.52	0.01

*Model R*^*2*^ *= 0.37; p <  0.001;* ^a^Mohr S, B, Gorham E, D, Garland C, F, Grant W, B, Garland F, C: Low Ultraviolet B and Increased Risk of Brain Cancer: An Ecological Study of 175 Countries. Neuroepidemiology 2010; 35:281–290. doi: 10.1159/000314350. ^b^Christenson E et al. [22]. ^c,d^ The World Bank. ^e^Institute for Health Metrics and Evaluation (IHME). ^f^ The Food and Agricultural Organization of the United Nations (FAO)

**Table 14:** Modeled 25(OH) D in association with incidence of CRC in 45–59 years of age, controlling for covariates

Covariate	Regression Coefficient	Standard Error	*t*	*p*-value
Modeled 25(OH) D, ng/ml^a^	−0.23	0.12	−1.92	0.06
Urbanization^b^	−0.02	0.07	−0.32	0.75
GDP^c^	< 0.001	< 0.001	2.05	0.04
Life Expectancy^d^	0.001	0.001	0.74	0.46
Smoking^e^	0.60	0.19	3.16	0.002
Animal Consumption^f^	0.29	0.06	4.57	< 0.001
Intercept	11.51	7.93	1.45	0.15

*Model R*^*2*^ *= 0.58; p <  0.001;* ^a^Mohr S, B, Gorham E, D, Garland C, F, Grant W, B, Garland F, C: Low Ultraviolet B and Increased Risk of Brain Cancer: An Ecological Study of 175 Countries. Neuroepidemiology 2010; 35:281–290. doi: 10.1159/000314350. ^b^Christenson E et al. [22]. ^c,d^ The World Bank. ^e^Institute for Health Metrics and Evaluation (IHME). ^f^ The Food and Agricultural Organization of the United Nations (FAO)

**Table 15:** Modeled 25(OH) D in association with incidence of CRC in 60–74 years of age, controlling for covariates

Covariate	Regression Coefficient	Standard Error	*t*	*p*-value
Modeled 25(OH) D, ng/ml^a^	−1.30	0.40	−3.28	0.001
Urbanization^b^	0.04	0.25	0.17	0.87
GDP^c^	0.001	< 0.001	2.12	0.04
Life Expectancy^d^	0.001	0.005	0.19	0.85
Smoking^e^	1.73	0.63	2.74	0.007
Animal Consumption^f^	0.86	0.21	4.09	< 0.001
Intercept	52.72	26.52	1.98	0.048

*Model R*^*2*^ *= 0.61; p < 0.001*; ^a^Mohr S, B, Gorham E, D, Garland C, F, Grant W, B, Garland F, C: Low Ultraviolet B and Increased Risk of Brain Cancer: An Ecological Study of 175 Countries. Neuroepidemiology 2010; 35:281–290. doi: 10.1159/000314350. ^b^Christenson E et al. [22]. ^c,d^ The World Bank. ^e^Institute for Health Metrics and Evaluation (IHME). ^f^ The Food and Agricultural Organization of the United Nations (FAO)

**Table 16:** Modeled 25(OH) D in association with incidence of CRC >/=75 years of age, controlling for covariates

Covariate	Regression Coefficient	Standard Error	*t*	*p*-value
Modeled 25(OH) D, ng/ml^a^	−1.79	0.63	−2.83	0.005
Urbanization^b^	0.27	0.40	0.68	0.50
GDP^c^	0.002	0.001	3.01	0.003
Life Expectancy^d^	0.001	0.007	0.11	0.91
Smoking^e^	1.66	1.01	1.64	0.10
Animal Consumption^f^	1.40	0.34	4.15	< 0.001
Intercept	75.33	42.31	1.78	0.08

*Model R*^*2*^ *= 0.62; p < 0.001;* ^a^Mohr S, B, Gorham E, D, Garland C, F, Grant W, B, Garland F, C: Low Ultraviolet B and Increased Risk of Brain Cancer: An Ecological Study of 175 Countries. Neuroepidemiology 2010; 35:281–290. doi: 10.1159/000314350. ^b^Christenson E et al. [22]. ^c,d^ The World Bank. ^e^Institute for Health Metrics and Evaluation (IHME). ^f^ The Food and Agricultural Organization of the United Nations (FAO)

## Appendix A

### List of excluded countries for which UVB estimates were unavailable

1. Cabo Verde
2. Côte d’Ivoire
3. France, Guadeloupe
4. France, La Réunion
5. France, Martinique
6. France, New Caledonia
7. French Guyana
8. French Polynesia
9. Gaza Strip and West Bank
10. Korea, Democratic Republic of
11. Korea, Republic of
12. Maldives
13. Montenegro
14. Republic of Moldova
15. Saint Lucia
16. Sao Tome and Principe
17. South Sudan
18. The former Yugoslav Republic of Macedonia
19. Timor-Leste

## Appendix B

### List of countries excluded from the multiple linear regression model

1. Bahrain
2. Bhutan
3. Burundi
4. Comoros
5. Congo, Democratic Republic of
6. Cuba
7. Djibouti
8. Equatorial Guinea
9. Eritrea
10. Guam
11. Libya
12. Papua New Guinea
13. Puerto Rico
14. Qatar
15. Singapore
16. Somalia
17. Swaziland
18. Syrian Arab Republic

## Appendix C

### List of the codes for the representation of names of countries (ISO 3166 standard)

1. AFG Afghanistan
2. AGO Angola
3. ALB Albania
4. ARE United Arab Emirates
5. ARG Argentina
6. ARM Armenia
7. ATA Antarctica
8. AUS Australia
9. AUT Austria
10. AZE Azerbaijan
11. BDI Burundi
12. BEL Belgium
13. BEN Benin
14. BFA Burkina Faso
15. BGD Bangladesh
16. BGR Bulgaria
17. BHR Bahrain
18. BHS Bahamas
19. BIH Bosnia and Herzegovina
20. BLR Belarus
21. BLZ Belize
22. BOL Bolivia
23. BRA Brazil
24. BRB Barbados
25. BRN Brunei Darussalam
26. BTN Bhutan
27. BWA Botswana
28. CAF Central African Republic
29. CAN Canada
30. CHE Switzerland
31. CHL Chile
32. CHN China
33. CMR Cameroon
34. COD Congo, Democratic Republic of the
35. COG Congo
36. COL Colombia
37. COM Comoros
38. CRI Costa Rica
39. CUB Cuba
40. CYP Cyprus
41. CZE Czechia
42. DEU Germany
43. DJI Djibouti
44. DNK Denmark
45. DOM Dominican Republic
46. DZA Algeria
47. ECU Ecuador
48. EGY Egypt
49. ERI Eritrea
50. ESP Spain
51. EST Estonia
52. ETH Ethiopia
53. FIN Finland
54. FJI Fiji
55. FRA France
56. GAB Gabon
57. GBR United Kingdom
58. GEO Georgia
59. GHA Ghana
60. GIN Guinea
61. GMB Gambia
62. GNB Guinea-Bissau
63. GNQ Equatorial Guinea
64. GRC Greece
65. GTM Guatemala
66. GUM Guam
67. GUY Guyana
68. HND Honduras
69. HRV Croatia
70. HTI Haiti
71. HUN Hungary
72. IDN Indonesia
73. IND India
74. IRL Ireland
75. IRN Iran
76. IRQ Iraq
77. ISL Iceland
78. ISR Israel
79. ITA Italy
80. JAM Jamaica
81. JOR Jordan
82. JPN Japan
83. KAZ Kazakhstan
84. KEN Kenya
85. KGZ Kyrgyzstan
86. KHM Cambodia
87. KWT Kuwait
88. LAO Laos
89. LBN Lebanon
90. LBR Liberia
91. LBY Libya
92. LKA Sri Lanka
93. LSO Lesotho
94. LTU Lithuania
95. LUX Luxembourg
96. LVA Latvia
97. MAR Morocco
98. MDG Madagascar
99. MEX Mexico
100. MLI Mali
101. MLT Malta
102. MMR Myanmar
103. MNG Mongolia
104. MOZ Mozambique
105. MRT Mauritania
106. MUS Mauritius
107. MWI Malawi
108. MYS Malaysia
109. NAM Namibia
110. NER Niger
111. NGA Nigeria
112. NIC Nicaragua
113. NLD Netherlands
114. NOR Norway
115. NPL Nepal
116. NZL New Zealand
117. OMN Oman
118. PAK Pakistan
119. PAN Panama
120. PER Peru
121. PHL Philippines
122. PNG Papua New Guinea
123. POL Poland
124. PRI Puerto Rico
125. PRT Portugal
126. PRY Paraguay
127. QAT Qatar
128. ROU Romania
129. RUS Russian Federation
130. RWA Rwanda
131. SAU Saudi Arabia
132. SDN Sudan
133. SEN Senegal
134. SGP Singapore
135. SLB Solomon Islands
136. SLE Sierra Leone
137. SLV El Salvador
138. SOM Somalia
139. SRB Serbia
140. SUR Suriname
141. SVK Slovakia
142. SVN Slovenia
143. SWE Sweden
144. SYR Syrian Arab Republic
145. TCD Chad
146. TGO Togo
147. THA Thailand
148. TJK Tajikistan
149. TKM Turkmenistan
150. TTO Trinidad and Tobago
151. TUN Tunisia
152. TUR Turkey
153. TZA Tanzania
154. UGA Uganda
155. UKR Ukraine
156. URY Uruguay
157. USA United States of America
158. UZB Uzbekistan
159. VEN Venezuela
160. VNM Viet Nam
161. VUT Vanuatu
162. WSM Samoa
163. YEM Yemen
164. ZAF South Africa
165. ZMB Zambia
166. ZWE Zimbabwe

## Abbreviations

CRC: Colorectal Cancer
UVB: Ultraviolet B
DINOMIT: Disjunction, Initiation, Natural selection, Overgrowth, Metastasis, Involution, Transition
25(OH)D: 25-hydroxyvitamin D
Vitamin D status: as assessed by serum 25(OH) D concentration
VDR: Vitamin D Receptor
BMI: Body Mass Index
WHS: Women’s Health Study
MEG3: Maternally expressed gene
GLOBOCAN: Global Cancer Database
NASA: National Aeronautics and Space Administration
ISCCP: International Satellite Cloud Climatology Project
GDP: Gross Domestic Product
PPP: Purchasing Power Parity
GHDx: Global Health Data Exchange
IHME: Institute for Health Metrics and Evaluation
FAO: Food and Agricultural Organization of the United Nations
QGIS: Quantum Geographic Information System
Choropleth map: Areas in the map are color coded to a variable.
