# Supplementary figures and images for: Could age increase the strength of inverse association between ultraviolet B exposure and colorectal cancer?
Source: BMC Public Health. 2021 Jul 5;21:1238. doi: 10.1186/s12889-021-11089-w (PMC8256562; doi:10.1186/s12889-021-11089-w)

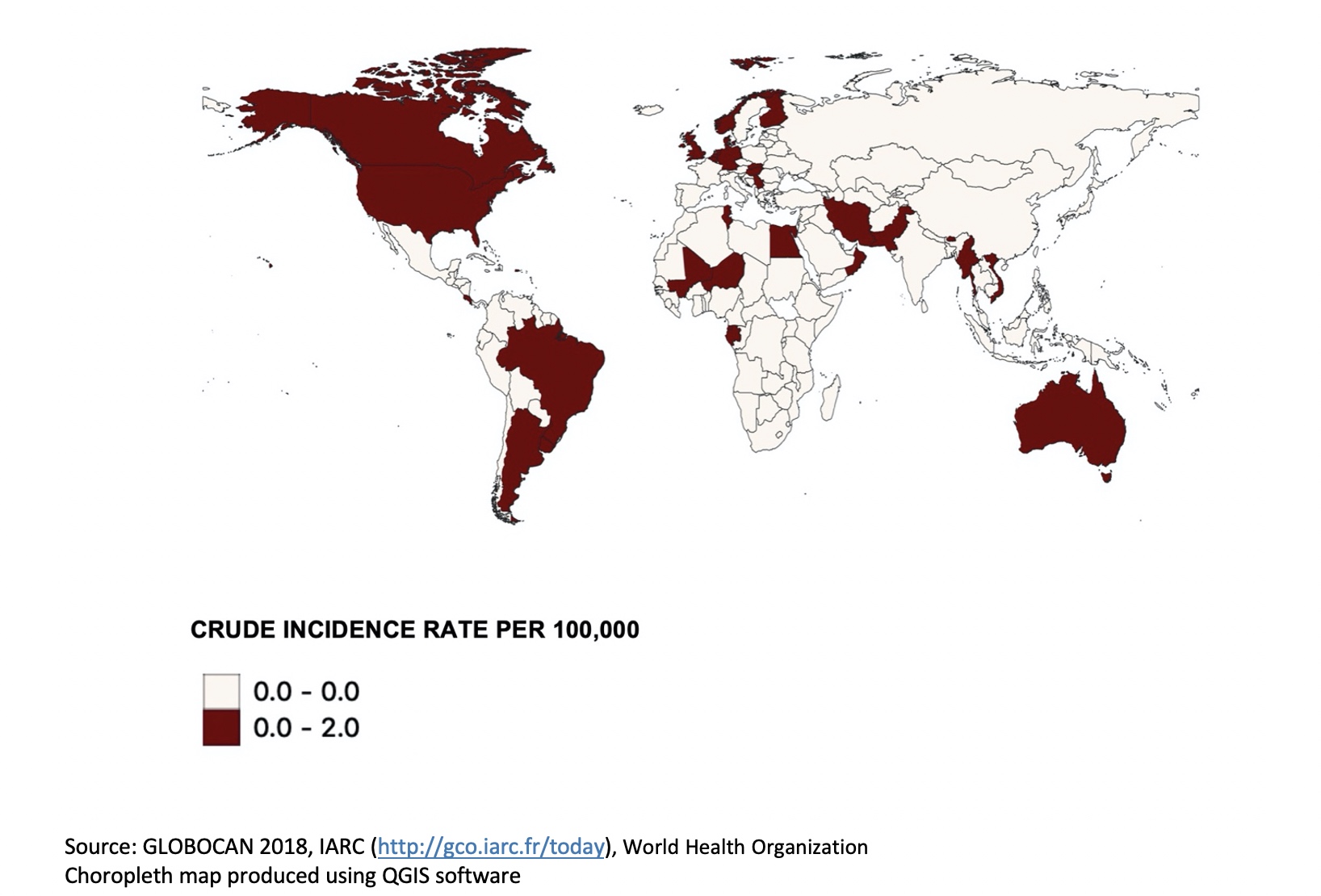

Supplement: Supplementary file 1 — Additional file 1: Figure S1. Colorectal cancer crude incidence rates, 15–29 years of age, all races, both sexes, 2018. [file 12889_2021_11089_MOESM1_ESM.jpg]

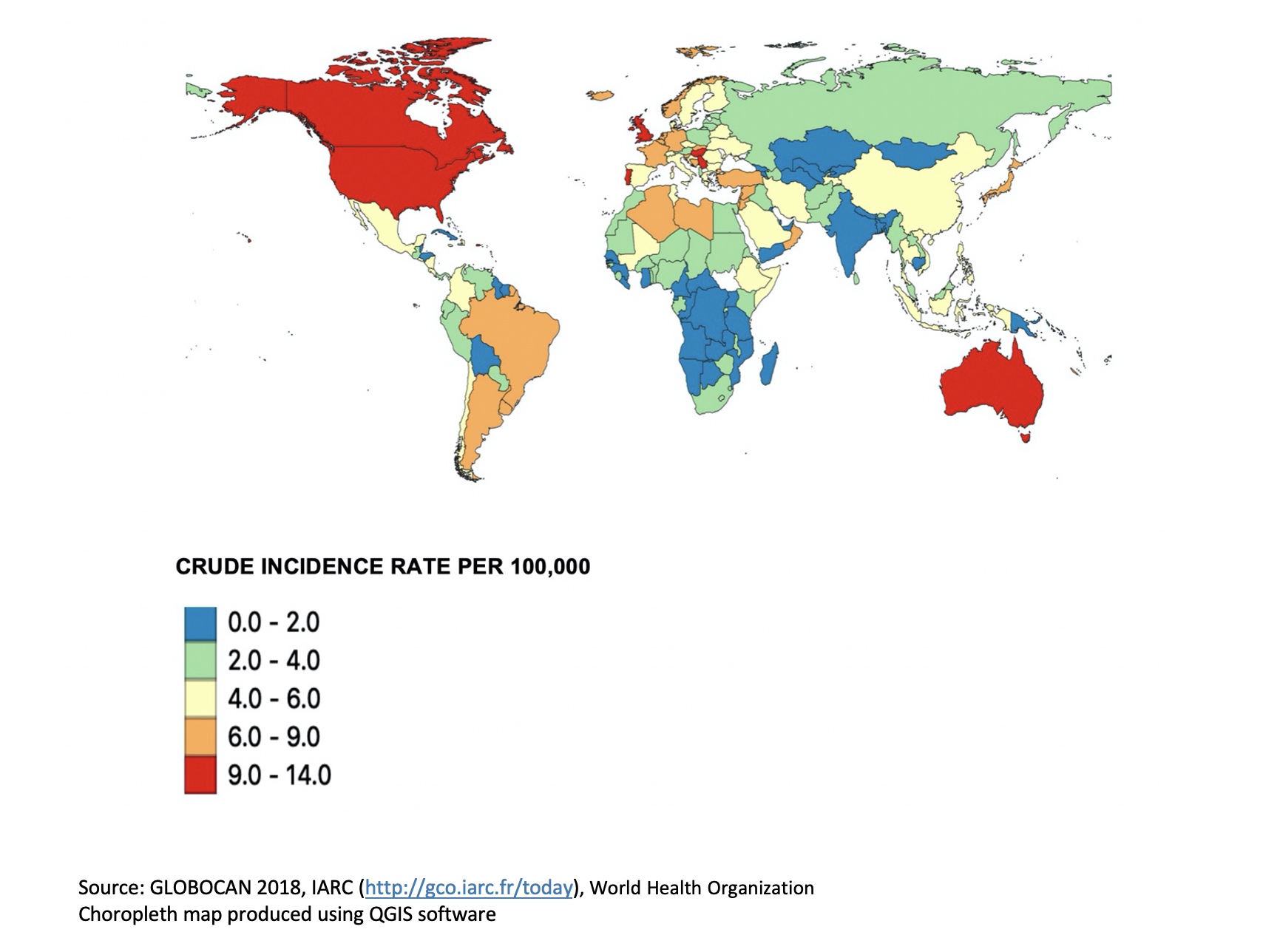

Supplement: Supplementary file 2 — Additional file 2: Figure S2. Colorectal cancer crude incidence rates, 30–44 years of age, all races, both sexes, 2018. [file 12889_2021_11089_MOESM2_ESM.jpg]

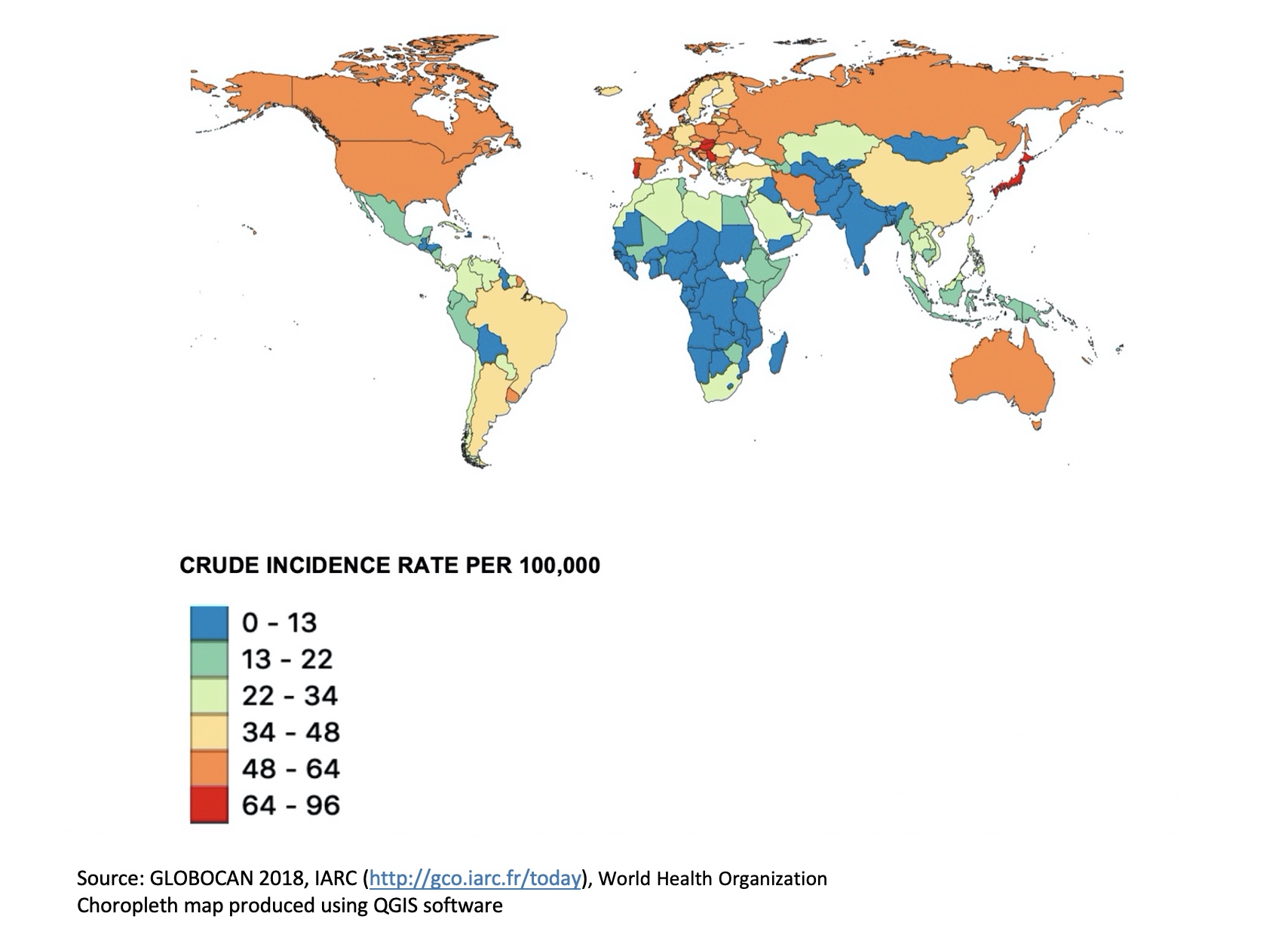

Supplement: Supplementary file 3 — Additional file 3: Figure S3. Colorectal cancer crude incidence rates, 45–59 years of age, all races, both sexes, 2018. [file 12889_2021_11089_MOESM3_ESM.jpg]

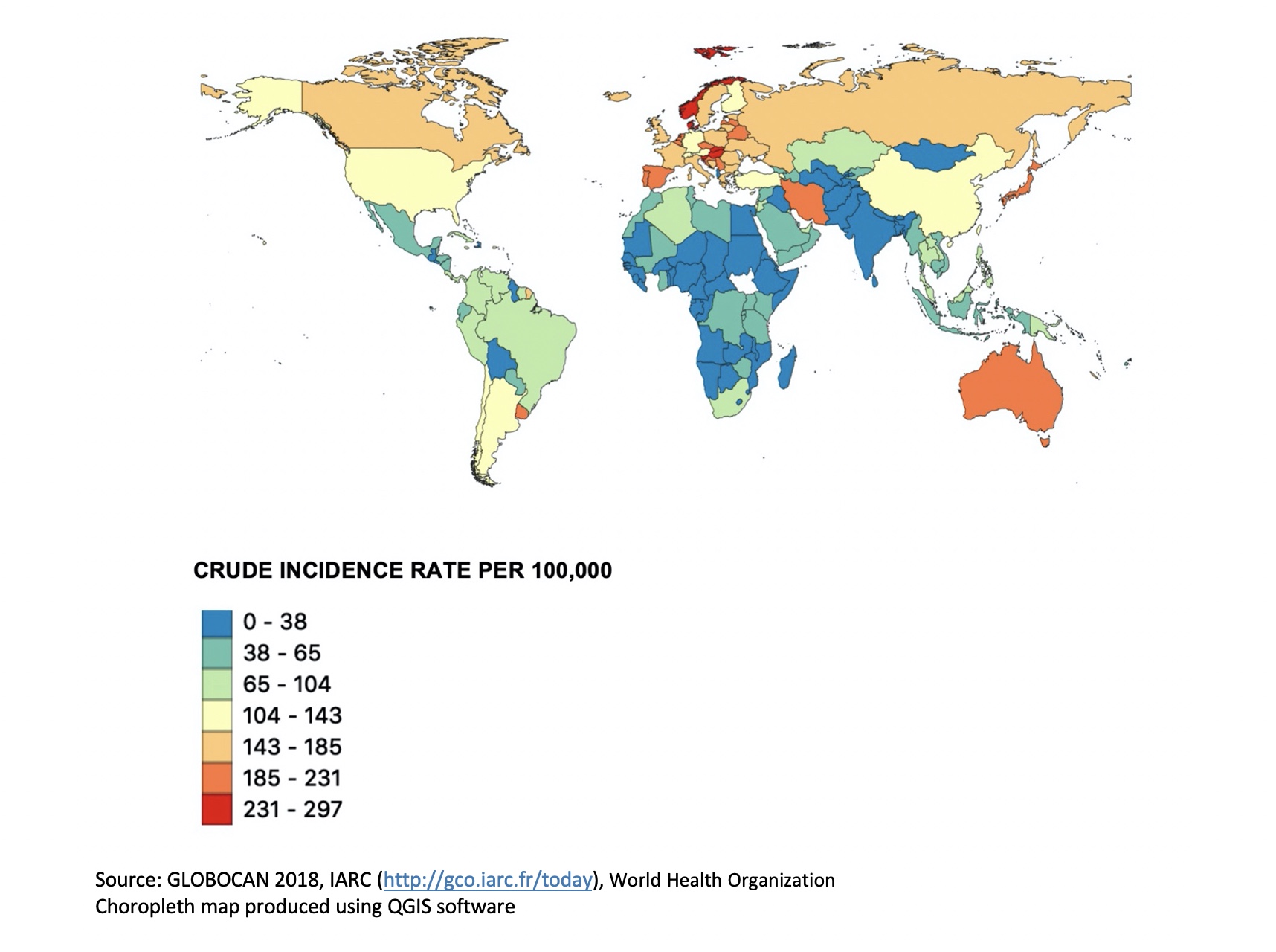

Supplement: Supplementary file 4 — Additional file 4: Figure S4. Colorectal cancer crude incidence rates, 60–74 years of age, all races, both sexes, 2018. [file 12889_2021_11089_MOESM4_ESM.jpg]

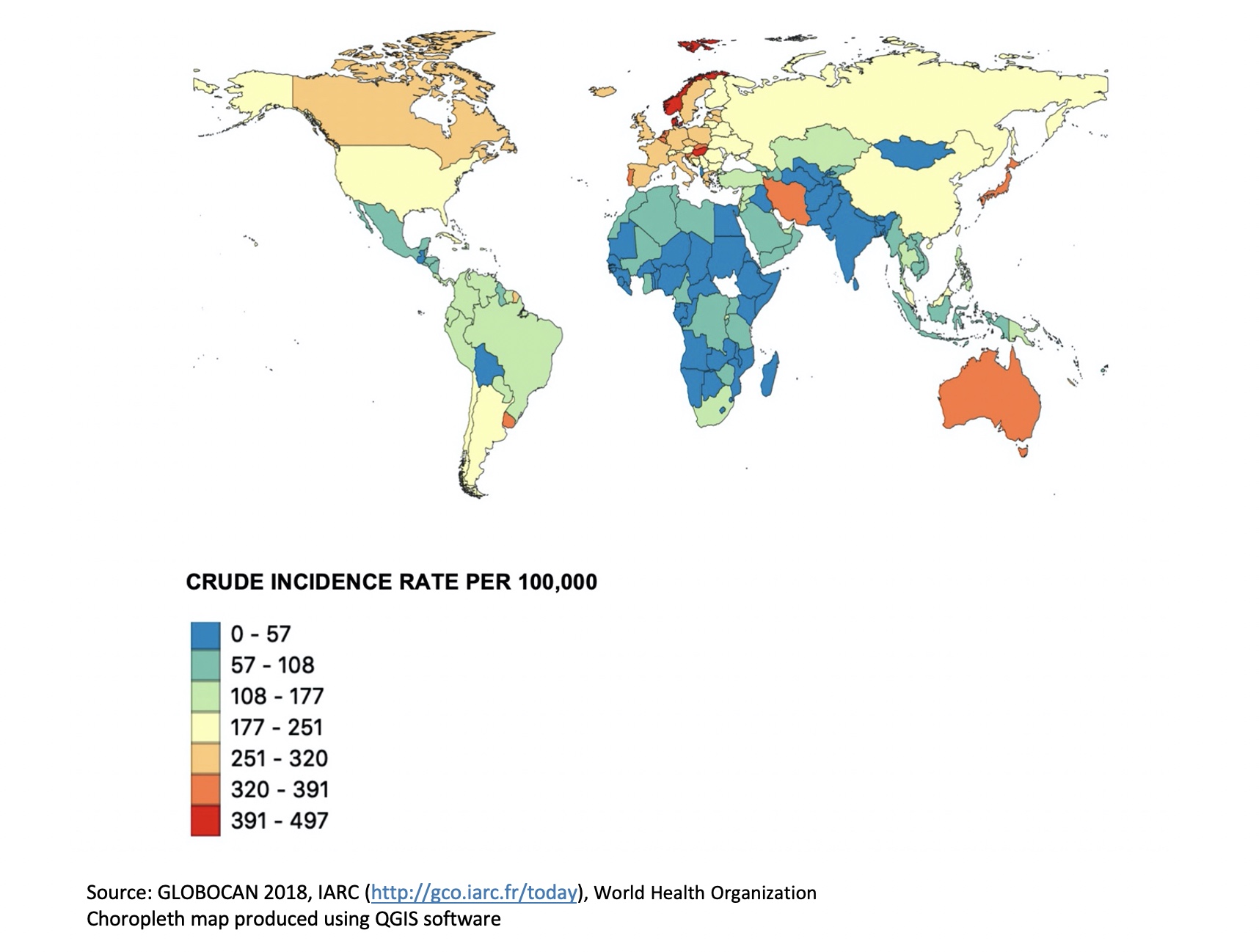

Supplement: Supplementary file 5 — Additional file 5: Figure S5. Colorectal cancer crude incidence rates, >/=75 years of age, all races, both sexes, 2018. [file 12889_2021_11089_MOESM5_ESM.jpg]
